# Complete genome sequence of *Actinosynnema mirum* type strain (101^T^)

**DOI:** 10.4056/sigs.21137

**Published:** 2009-07-20

**Authors:** Miriam Land, Alla Lapidus, Shanmugam Mayilraj, Feng Chen, Alex Copeland, Tijana Glavina Del Rio, Matt Nolan, Susan Lucas, Hope Tice, Jan-Fang Cheng, Olga Chertkov, David Bruce, Lynne Goodwin, Sam Pitluck, Manfred Rohde, Markus Göker, Amrita Pati, Natalia Ivanova, Konstantinos Mavromatis, Amy Chen, Krishna Palaniappan, Loren Hauser, Yun-Juan Chang, Cynthia C. Jeffries, Thomas Brettin, John C. Detter, Cliff Han, Patrick Chain, Brian J. Tindall, Jim Bristow, Jonathan A. Eisen, Victor Markowitz, Philip Hugenholtz, Nikos C. Kyrpides, Hans-Peter Klenk

**Affiliations:** 1 DOE Joint Genome Institute, Walnut Creek, California, USA; 2 Oak Ridge National Laboratory, Oak Ridge, Tennessee, USA; 3 DSMZ - German Collection of Microorganisms and Cell Cultures GmbH, Braunschweig, Germany; 4 Microbial Type Culture Collection, Institute of Microbial Technology, Chandigarh, India; 5 Los Alamos National Laboratory, Bioscience Division, Los Alamos, New Mexico USA; 6 HZI - Helmholtz Centre for Infection Research, Braunschweig, Germany; 7 Biological Data Management and Technology Center, Lawrence Berkeley National Laboratory, Berkeley, California, USA; 8 Lawrence Livermore National Laboratory, Livermore, California, USA; 9 University of California Davis Genome Center, Davis, California, USA

**Keywords:** Synnemata, motile spores, soluble pigments, mesophile, aerobic, aerial and substrate mycelium, nocardicin A producer, *Actinosynnemataceae*

## Abstract

*Actinosynnema mirum* Hasegawa *et al*. 1978 is the type species of the genus, and is of phylogenetic interest because of its central phylogenetic location in the *Actino-synnemataceae,* a rapidly growing family within the actinobacterial suborder *Pseudo-nocardineae*.* A. mirum* is characterized by its motile spores borne on synnemata and as a producer of nocardicin antibiotics. It is capable of growing aerobically and under a moderate CO_2_ atmosphere. The strain is a Gram-positive, aerial and substrate mycelium producing bacterium, originally isolated from a grass blade collected from the Raritan River, New Jersey. Here we describe the features of this organism, together with the complete genome sequence and annotation. This is the first complete genome sequence of a member of the family *Actinosynnemataceae*, and only the second sequence from the actinobacterial suborder *Pseudonocardineae*. The 8,248,144 bp long single replicon genome with its 7100 protein-coding and 77 RNA genes is part of the *** G****enomic* *** E****ncyclopedia of* *** B****acteria and* *** A****rchaea * project.

## Introduction

**Figure 1:**
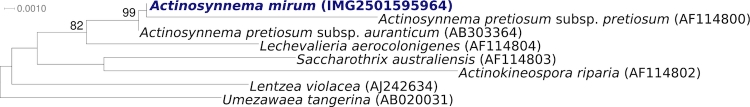
Phylogenetic tree highlighting the position of *A. mirum* 101^T^ relative to all type strains of the genus and to the type strains of the type species of all other genera within the family. The tree was inferred from 1,491 aligned characters [2,3] of the 16S rRNA gene sequence under the maximum likelihood criterion [4] and rooted in accordance with current actinobacterial taxonomy. The branches are scaled in terms of the expected number of substitutions per site. Numbers above branches are support values from 1,000 bootstrap replicates if larger than 60%. Lineages with a type strain genome-sequencing project registered in GOLD [5] are printed in blue; published genomes in bold.

Strain 101^T^ (DSM 43827 = ATCC 29888 = NBRC 14064, and other culture collections) is the type strain of *Actinosynnema mirum*, which is the type species of the genus *Actinosynnema* [1] (Figure 1). *A. mirum* was described by Hasegawa *et al*. in 1978 [1] as an aerobic actinobacterium which forms synnemata (compacted groups of erect hyphae which bear conidia) with zoospores [1]. The organism is of interest due to its position in the tree of life where the small genus *Actino-synnema*, currently comprising only two species, is located on a rather long branch within the rapidly growing actinobacterial suborder *Pseudo-nocardineae* [6]. We here present a summary classification and a set of features for *A. mirum* strain 101^T^ (Table 1), together with the description of the complete genomic sequencing and annotation.

**Table 1:** Classification and general features of *A. mirum* 101^T^ in accordance with the MIGS recommendations [7]

MIGS ID	Property	Term	Evidence code
	Current classification	Domain *Bacteria* Phylum *Actinobacteria* Class *Actinobacteria* Order *Actinomycetales* Suborder *Pseudonocardineae* Family *Actinosynnemataceae* Genus *Actinosynnema* Species *Actinosynnema mirum* Type strain 101	TAS [6] TAS [6] TAS [6] TAS [8] TAS [1] TAS [1]
	Gram stain	positive	TAS [1]
	Cell shape	hyphae, aerial and substrate mycelium	TAS [1]
	Motility	cells nonmotile; spores motile	TAS [1]
	Sporulation	sporulating	TAS [1]
	Temperature range	mesophilic	TAS [1]
	Optimum temperature	10-30°C	TAS [1]
	Salinity	no growth at 5g NaCl/l	TAS [1]
MIGS-22	Oxygen requirement	essentially aerobic; moderate growth under CO_2_ atmosphere	TAS [1]
	Carbon source	glucose, maltose, mannose, cellobiose	TAS [1]
	Energy source	chemoorganotrophic	TAS [1]
MIGS-6	Habitat	soil, river side	TAS [1]
MIGS-15	Biotic relationship	free-living	NAS
MIGS-14	Pathogenicity	none	NAS
	Biosafety level	1	TAS [9]
	Isolation	grass blade	TAS [1]
MIGS-4	Geographic location	Raritan River, New Jersey	TAS [1]
MIGS-5	Sample collection time	September 1976	TAS [1]
MIGS-4.1 MIGS-4.2	Latitude – Longitude	40.491816, -74.322087	NAS
MIGS-4.3	Depth	not reported	
MIGS-4.4	Altitude	not reported	

Evidence codes - IDA: Inferred from Direct Assay (first time in publication); TAS: Traceable Author Statement (i.e., a direct report exists in the literature); NAS: Non-traceable Author Statement (i.e., not directly observed for the living, isolated sample, but based on a generally accepted property for the species, or anecdotal evidence). These evidence codes are from the GeneOntology project [10]. If the evidence code is IDA, then the property was directly observed for a live isolate by one of the authors or an expert mentioned in the acknowledgements.

## Classification and features

No closely related cultivated strains are known from the literature that can be linked to the species *A. mirum.* Curiously, the 16S rRNA gene sequences of the type strains from the two subspecies within the second species of the genus *Actinosynnema*, *A. pretiosum* subsp. *auranticum* (AB303364) and *A. pretiosum* subsp. *pretiosum* (AB303365) [11], seem to have an equally or even higher degree of similarity to the 16S rRNA gene sequence derived from the genome sequence reported here than the previously reported gene sequences of strain 101^T^ (see Figure 1). None of the phylotypes reported from environmental screenings or genomic surveys could be linked to *A. mirum* with a convincing degree of sequence similarity (maximal observed degree of similarity 92%; status June 2009).

**Figure 2:**
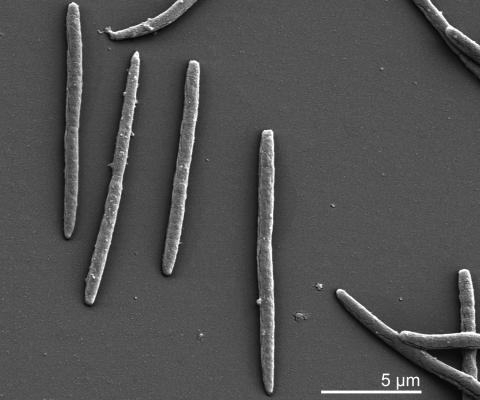
Scanning electron micrograph (SEM) of *A. mirum* 101^T^. More SEMs of *A. mirum* can be found in the CompendiumoftheActinobacteria, by Joachim M. Wink, University of Braunschweig.

*A. mirum* strain 101^T^ cells are non-motile with fine hyphae which form aerial and substrate mycelia. Both the aerial and substrate mycelia are about 0.5 to 1.0 µm in diameter. Aerial mycelia are long branching hyphae, white to pale yellow in color (Figure 2). The substrate mycelia are also long, branching hyphae, white to yellowish orange, and penetrate into the agar medium and form synnemata [1]. Cells stain Gram-positive and are non-acid fast [1].

*A. mirum* is capable of producing a yellowish-brown soluble pigment on tyrosine agar and a pale greenish pigment on oatmeal agar [1]. Capable of hydrolyzing starch, casein, tyrosine and gelatin, but not xanthine, hypoxanthine, adenine and urea [1]; produces nitrate reductase and phosphatase. Positive for utilization of tartrate, pyruvate, lactate and malate, but negative for benzoate, acetate, citrate and succinate [1]. Acid is produced aerobically from fructose, lactose, maltose, D-mannitol, L-arabinose, D-melibiose, D-mannose, L-rhamnose, xylose, dextrin, galactose, glucose, trehalose, raffinose, starch, sucrose, cellobiose, glycogen and adonitol, but not from inositol, sorbitol, D-ribose, salicin, inulin, glycerol, dulcitol, erythritol, α-methyl-D-glucoside and α-methyl-D-mannoside. *A. mirum* is a producer of nocardicin antibiotics [12] and inhibits the growth of several Gram-positive bacteria including: *Bacillus megaterium*, *Sarcina lutea*, *Mycobacterium smegmatis*; as well as the filamentous fungi, *Aspergillus niger*, *Penicillium notatum* and the yeasts, *Saccharomyces cerevisiae* and *Candida tropicalis*.

Figure 1 shows the phylogenetic neighborhood of *A. mirum* strain 101^T^ in a 16S rRNA based tree. The sequences of the five 16S rRNA genes in the *A. mirum* genome differ by no more than one nucleotide (nt) from each other, and by up to six nts from the previously reported reference sequences derived from NBRC 14064 (AF328679) and from DSM 43827 (X84447). The differences between the genome data and the previously reported 16S rRNA gene sequence are probably due to sequencing errors in the previously reported sequence data.

### Chemotaxonomy

The peptidoglycan of *A. mirum* contains *meso*-diaminopimelic acid in addition to alanine, glutamic acid and glucosamine. Galactose and mannose are present in the cell wall sugars, whereas madurose is absent. Cell wall type III has been detected, as well as whole-cell sugar pattern of type C [1]. The fatty acid pattern of strain 101^T^ is dominated by saturated straight chain acids, C_17:0_ (15.2%), C_16:0_ (4.8%), C_15:0_ (2.6%), and branched chain acids, anteiso-(ai-)C_13:0_ (11.6%), ai-C_15:0_ (5.9%), ai-C_17:0_ (4.5%), ai-C_11:0_ (2.3%), and iso-(i-)C_12:0_ (11.3%), i-C_16:0_ (7.5%), i-C_14:0_ (3.5%), i-C_15:0_ (2.1%), i-C_11:0_ (1.5%). Unsaturated straight chain acids play only a limited role: C_17:1 cis9_ (11.3%), and C_16:1 cis9_ (3.4%) are present, whereas unsaturated branched chain fatty acids are absent. Minor amounts of hydroxylated fatty acids were detected: C_16:1_ 2OH (1.0%), ai-C_15:0_ 2OH (0.9%), and C_15:0_ 3OH (0.5%) [Cellular fatty acids data from RM Kroppenstedt, DSMZ, unpublished]. The published literature on the fatty acid patterns is, however, contradictory, with Hasegawa *et al.* [11], and Yassin e*t al* [13]. emphasizing the presence of branched chain fatty acids (including a 10-methyl C18:0), but neither unsaturated nor hydroxylated fatty acids are reported. The major polar lipids present are: diphosphatidylglycerol (DPG), phosphatidylethanolamine (PE), phosphatidyl inositol mannosides (PIM) and phosphatidylinositol (PI) [13]. Hydroxy-phosphatidylethanolamine (OH-PE) has been reported by some authors [14,15], but not by others [8,13]. MK-9(H4) and MK-9(H6) are the predominant menaquinones [13].

## Genome sequencing and annotation

### Genome project history

This organism was selected for sequencing on the basis of its phylogenetic position, and is part of the *** G****enomic* *** E****ncyclopedia of* *** B****acteria and* *** A****rchaea * project. The genome project is deposited in the Genomes OnLine Database [5] and the complete genome sequence in GenBank (CP001630). Sequencing, finishing and annotation were performed by the DOE Joint Genome Institute (JGI). A summary of the project information is shown in Table 2.

**Table 2:** Genome sequencing project information

MIGS ID	Property	Term
MIGS-31	Finishing quality	Finished
MIGS-28	Libraries used	Two genomic libraries: 8kb pMCL200 and fosmid pcc1Fos Sanger libraries. One 454 pyrosequence standard library
MIGS-29	Sequencing platforms	ABI3730, 454 GS FLX
MIGS-31.2	Sequencing coverage	8.9x Sanger; 20x pyrosequence
MIGS-30	Assemblers	Newbler version 1.1.02.15, phrap
MIGS-32	Gene calling method	Prodigal
	Genbank ID	CP001630
	Genbank Date of Release	not available
	GOLD ID	Gc01024
	NCBI project ID	19705
	Database: IMG-GEBA	2501533214
MIGS-13	Source material identifier	DSM 43827
	Project relevance	Tree of Life, GEBA

### Growth conditions and DNA isolation

*A. mirum* strain 101^T^, DSM 44827, was grown in DSMZmedium535 (GYM *Streptomyces* Medium at 28°C. DNA was isolated from 1-1.5 g of cell paste using Qiagen Genomic 500 DNA Kit (Qiagen, Hilden, Germany) with a modified lysis buffer (1 ml achromopeptidase and 0.5 ml lysostaphin added) and one hour incubation at 37°C.

### Genome sequencing and assembly

The genome was sequenced using a combination of Sanger and 454 sequencing platforms. All general aspects of library construction and sequencing performed at the JGI can be found on the JGIwebsite. 454 Pyrosequencing reads were assembled using the Newbler assembler version 1.1.02.15 (Roche). Large Newbler contigs were broken into 10,493 overlapping fragments of 1,000 bp and entered into assembly as pseudo-reads. The sequences were assigned quality scores based on Newbler consensus q-scores with modifications to account for overlap redundancy and to adjust inflated q-scores. A hybrid 454/Sanger assembly was made using the phrap assembler (High Performance Software, LLC). Possible mis-assemblies were corrected with Dupfinisher or transposon bombing of bridging clones [16]. Gaps between contigs were closed by editing in Consed, custom primer walk or PCR amplification. 1,564 Sanger finishing reads were produced to close gaps and to raise the quality of the finished sequence. The error rate of the completed genome sequence is less than 1 in 100,000. Together all sequence types provided 28.9x coverage of the genome. The final assembly contains 105,508 Sanger reads in addition to the 454 based pseudo reads.

### Genome annotation

Genes were identified using Prodigal [17] as part of the Oak Ridge National Laboratory genome annotation pipeline, followed by a round of manual curation using the JGI GenePRIMP pipeline [18]. The predicted CDSs were translated and used to search the National Center for Biotechnology Information (NCBI) nonredundant database, UniProt, TIGRFam, Pfam, PRIAM, KEGG, COG, and InterPro databases. Additional gene prediction analysis and functional annotation was performed within the IntegratedMicrobialGenomes (IMG-ER) platform [19].

## Genome properties

**Figure 3:**
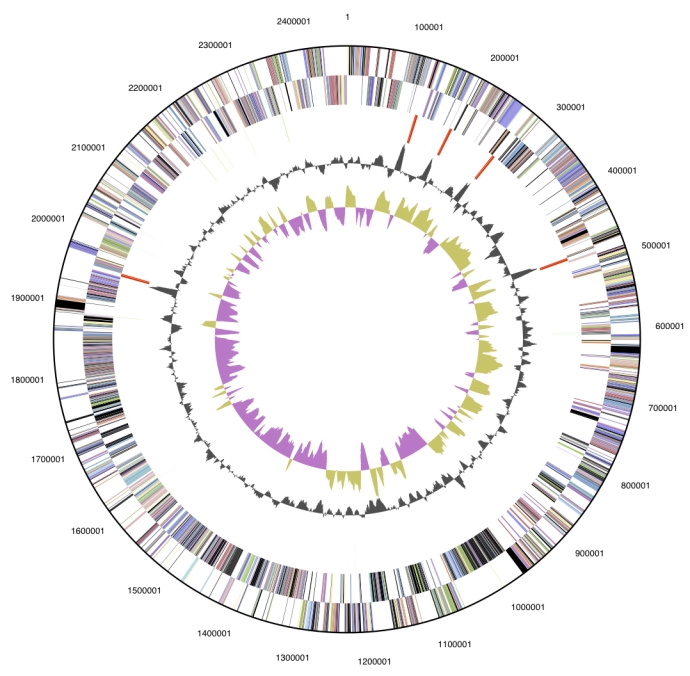
Graphical circular map of the genome. From outside to the center: Genes on forward strand (color by COG categories), Genes on reverse strand (color by COG categories), RNA genes (tRNAs green, rRNAs red, other RNAs black), GC content, GC skew.

The genome is 8,248,144 bp long and comprises one circular chromosome with a 73.7% GC content (Table 3 and Figure 3). Of the 7,174 genes predicted, 7100 were protein coding genes, and 74 RNAs., One hundred and eight four pseudogenes were also identified. The majority of genes (67.3%) of the genes were assigned a putative function while the remaining ones were annotated as hypothetical proteins. The prop-erties and the statistics of the genome are summarized in Table 3. The distribution of genes into COGs functional categories is presented in Table 4.

**Table 3:** Genome Statistics

Attribute	Value	% of Total
Genome size (bp)	8,248,144	
DNA Coding region (bp)	7,331,694	88.89%
DNA G+C content (bp)	6,079,614	73.71%
Number of replicons	1	
Extrachromosomal elements	0	
Total genes	7174	
RNA genes	74	1.07%
rRNA operons	5	
Protein-coding genes	7100	98.93%
Pseudo genes	184	2.56%
Genes with function prediction	4835	67.37%
Genes in paralog clusters	1404	19.56%
Genes assigned to COGs	4487	62.52%
Genes assigned Pfam domains	4849	67.56%
Genes with signal peptides	1722	23.99%
Genes with transmembrane helices	1590	21.15%
CRISPR repeats	0	

**Table 4:** Number of genes associated with the 21 general COG functional categories

Code	Value	%	Description
J	182	2.6	Translation, ribosomal structure and biogenesis
A	2	0.0	RNA processing and modification
K	607	8.5	Transcription
L	173	2.4	Replication, recombination and repair
B	2	0.0	Chromatin structure and dynamics
D	34	0.5	Cell cycle control, mitosis and meiosis
Y	0	0.0	Nuclear structure
V	96	1.4	Defense mechanisms
T	389	5.5	Signal transduction mechanisms
M	210	3.0	Cell wall/membrane biogenesis
N	45	0.6	Cell motility
Z	1	0.0	Cytoskeleton
W	0	0.0	Extracellular structures
U	46	0.6	Intracellular trafficking and secretion
O	149	2.1	Posttranslational modification, protein turnover, chaperones
C	306	4.3	Energy production and conversion
G	441	6.2	Carbohydrate transport and metabolism
E	425	6.0	Amino acid transport and metabolism
F	108	1.5	Nucleotide transport and metabolism
H	223	3.1	Coenzyme transport and metabolism
I	226	3.2	Lipid transport and metabolism
P	241	3.4	Inorganic ion transport and metabolism
Q	265	3.7	Secondary metabolites biosynthesis, transport and catabolism
R	670	9.4	General function prediction only
S	328	4.6	Function unknown
-	2613	36.8	Not in COGs
